# Real-time computed tomography fluoroscopy-guided solitary lung tumor model in a rabbit

**DOI:** 10.1371/journal.pone.0179220

**Published:** 2017-06-13

**Authors:** Byeong Hyeon Choi, Hwan Seok Young, Yu Hua Quan, Jiyun Rho, Jae Seon Eo, Kook Nam Han, Young Ho Choi, Kim Hyun Koo

**Affiliations:** 1 Department of Biomedical Sciences, College of Medicine, Korea University, Seoul, Korea; 2 Department of Thoracic and Cardiovascular Surgery, Korea University Guro Hospital, College of Medicine, Korea University, Seoul, Korea; 3 Department of Radiology, Korea University Guro Hospital, College of Medicine, Korea University, Seoul, Korea; 4 Department of Nuclear Medicine, Korea University Guro Hospital, College of Medicine, Korea University, Seoul, Korea; Central South University, The Third Xiang Ya Hospital, CHINA

## Abstract

Preclinical studies of lung cancer require suitable large-animal models to allow evaluation and development of surgical and interventional techniques. We assessed the feasibility and safety of a novel rabbit lung cancer model of solitary tumors, in which real-time computed tomography fluoroscopy is used to guide inoculation of VX2 carcinoma single-cell suspensions. Thirty-eight rabbits were divided into four groups according to the volume of the VX2 tissue or cell suspension, the volume of lipiodol, the volume of Matrigel, and the injection needle size. The mixtures were percutaneously injected into rabbit lungs under real-time computed tomography fluoroscopy guidance. Two weeks later, VX2 lung carcinomas were confirmed via positron emission tomography/computed tomography, necropsy, and histology. Real-time computed tomography fluoroscopy allowed the precise inoculation of the tumor cell suspensions containing lipiodol, while the use of Matrigel and a small needle prevented leakage of the suspensions into the lung parenchyma. Solitary lung tumors were successfully established in rabbits (n = 22) inoculated with single-cell suspensions (150 μL), lipiodol (150 μL), and Matrigel (150 μL) using a 26-gauge needle. This combination was determined to be optimal. Pneumothorax was observed in only two of the 38 rabbits (5.3%), both of which survived to the end of the study without any intervention. Real-time computed tomography fluoroscopy-guided inoculation of VX2 single-cell suspensions with lipiodol and Matrigel using a small needle is an easy and safe method to establish solitary lung tumors in rabbits.

## Introduction

Lung cancer has a poor prognosis and is one of the leading causes of cancer mortality worldwide. The 5-year survival rate of approximately 18% is much lower than the rates for breast cancer (91%) and thyroid cancer (98%) [1]. However, the 5-year overall survival rate is almost 50% higher for stage I non-small cell lung cancer (NSCLC), which is localized and shows no metastasis to the lymph nodes or other organs [2], and it is around 90% for NSCLCs ≤1 cm [3]. Therefore, early detection is considered the most important factor associated with good prognosis after treatment for NSCLC.

Surgical resection (i.e., lobectomy) is the treatment of choice for patients with early-stage NSCLC, as well as the only treatment that offers any prospect of cure or long-term survival. However, limited resection, such as segmentectomy or wedge resection, is an alternative option for patients with stage Ia NSCLC or NSCLCs with small amounts of peripherally located ground-glass opacity. Recent increases in the detection rate of early-stage NSCLCs, resulting from computed tomography (CT) screening programs, have generated interest in limited resection as an alternative to lobectomy that better maintains quality of life, especially in patients with poor pulmonary reserve. Another minimally invasive procedure is video-assisted thoracoscopic surgery (VATS), which is now routinely used for lung resection owing to recent technical improvements. VATS is associated with less postoperative pain and a shorter recovery period than is found with conventional open thoracotomy [4]. Despite their numerous advantages, however, minimally invasive procedures may result in incomplete removal of the cancerous pulmonary lesions, resulting in local recurrence [5, 6]. Specifically, in cases of tumors that are small or deeply located, and thus impalpable or invisible, it is difficult to achieve exact identification of resection margins via such procedures.

Visualization of a lung tumor during surgery allows precise delineation of resection margins and can be accomplished by using lipiodol, microcoils, fluorescence imaging, or the hookwire technique [4, 7–12]. Other methods include near-infrared fluorescence imaging, a novel image-guided surgical procedure that involves peripheral injection of indocyanine green [13], and molecular imaging with targeted contrast agents [14, 15]. Although image-guided surgery holds promise for precise lung cancer surgery, the optimal fluorescent or molecular agents for passive accumulation or active targeting remain to be identified.

Preclinical studies of lung cancer require appropriate large-animal models to allow evaluation and development of surgical techniques, such as those used in minimally invasive procedures. In the past 40 years, many lung cancer models have involved surgical implantation of tumor cells into small rodents [16, 17]. However, invasive implantation is associated with complications such as pneumothorax and pleural dissemination, and small animals are generally unsuitable for surgical studies.

The rabbit VX2 squamous tumor model is a longstanding model used in preclinical oncology studies. Rabbits are inexpensive, easy to manage in a laboratory setting, and have an organ system similar to that of humans. This large-animal tumor model can be used for imaging, as well as for development of interventional surgical instruments and surgical skills, because VX2 tumors grow well in a number of organs, including the liver, kidneys, brain, esophagus, and lungs [18–22]. Till date, rabbit lung cancer models have involved inoculation of invasive tumor cells [23]; however, inoculation is inconvenient as it requires mini-thoracotomy, and this procedure may lead to pneumothorax or unexpected infections. The recently introduced bronchoscopic VX2 inoculation is less invasive than traditional inoculation [17, 19], but insertion of the ultrathin bronchoscope and bronchoscopic needles into the narrow rabbit bronchus is challenging. Furthermore, animals must be maintained under general anesthesia during the procedure via mechanical ventilation using a prepared bronchoscopic accessory [22, 24].

Previous studies of lung cancer have reported CT-guided inoculations of VX2 tumor cell suspensions [25–27]. However, the success rate of such inoculations in lung cancer models of solitary tumors was low because injection of the suspension into the lung parenchyma was difficult to confirm in real time. Therefore, we developed a simple, but more effective, solitary lung tumor model that involved real-time CT fluoroscopy-guided inoculation of VX2 single-cell suspensions containing radiopaque dye and Matrigel into rabbits, with no associated complications of pneumothorax or pleural seeding. The present study evaluated the feasibility and safety of this novel rabbit lung cancer model.

## Materials and methods

### Animals and animal care

All study procedures, including animal care and handling, were approved by the Institutional Animal Care & Use Committee of Korea University. A total of 38 female New Zealand White rabbits (DooYeol Biotech Co. Ltd., Seoul, Korea), weighing approximately 2.5–3 kg each, were used in this study. To help the rabbits adapt to their environment, they were housed in individual cages with freely available food and water for 1–2 weeks, in accordance with our humane animal care protocol.

### VX2 carcinoma cell harvest

Donor rabbits with VX2 carcinomas in their hind limbs were used to maintain and propagate VX2 tumors. The rabbits were anesthetized via intramuscular injection of 10 mg/kg tiletamine-zolazepam (Zoletil 50; Virbac Korea Inc., Seoul, Korea) and 5 mg/kg xylazine (Rompun™; Bayer Korea Inc., Seoul, Korea). The hind limbs were shaved and sterilized with povidone-iodine, and tissue fragments of the VX2 carcinomas were excised using a surgical blade. For tumor transplantation, the VX2 tissue was washed with 3 mL phosphate-buffered saline (PBS) in a cell reservoir, and any surrounding tissue or necrotic tumor tissue was removed.

### Suspension optimization

We evaluated the effect of the following on successful formation of a localized single lung tumor: VX2 carcinoma cell number; volume of radiopaque dye (Lipiodol Ultra-Fluid; Guerbet Korea, Seoul, Korea) used for real-time CT-guided injection; volume of Matrigel (BD Bioscience, Beit-Ha’Emek, Israel) used to prevent suspension leaks; and size of the injection needle.

Four groups of rabbits were included in the study. Each group received a different preparation of VX2 tumor tissue or cells and other additives via injection into a single lung lobe (Table 1). Group 1 (n = 5) was injected with a 500 μL suspension of minced VX2 tumor tissue (0.5 g) using an 18-gauge needle. Group 2 (n = 5) was injected with a 400 μL suspension of unfiltered minced VX2 carcinoma tissue (0.5 g) and 100 μL lipiodol using a 20-gauge needle. For group 3 (n = 6), VX2 tumor tissue was finely minced using microdissecting scissors and isolated in 5 mL PBS; a 100-μm cell strainer was then used to obtain a single-cell suspension. The filtrate was centrifuged at 1200 rpm for 3 min at room temperature and resuspended in PBS at a concentration of 1 × 10^7^ cells/mL. The suspension (150 μL) was mixed with 150 μL lipiodol and 150 μL Matrigel and injected into the rabbits using a 20-gauge needle. Group 4 (n = 22) was injected with a mixture of 150 μL VX2 tumor cell suspension filtered through a 100-μm cell strainer, 150 μL lipiodol, and 150 μL Matrigel using a 26-gauge needle (KOVAX-SYRINGE 1ml, Korea Vaccine Co. ltd, Seoul, Korea).

**Table 1:** Different suspension compositions in the rabbit lung tumor model.

Group (n)	Volume (μL)			Needle size (gauge)
	Suspended cancer tissue or cells	Lipiodol	Matrigel	
1 (5)	500	0	0	18
2 (5)	400	100	0	20
3 (6)	150	150	150	20
4 (22)	150	150	150	26

### Real-time CT fluoroscopy-guided VX2 injection

Rabbits were anesthetized via injection of a mixture of tiletamine-zolazepam and xylazine, and their lateral chest regions were shaved.

Injection of the VX2 tumor/Matrigel/lipiodol mixtures was performed by board-certified radiologists with experience in CT-guided interventional techniques. Rabbits were placed in the gantry of the CT unit (Brilliance 64; Philips, Amsterdam, The Netherlands) in a position that allowed a safe access route for insertion of the needle. Throughout the procedure, real-time images were obtained via CT fluoroscopy.

**Fig 1:**
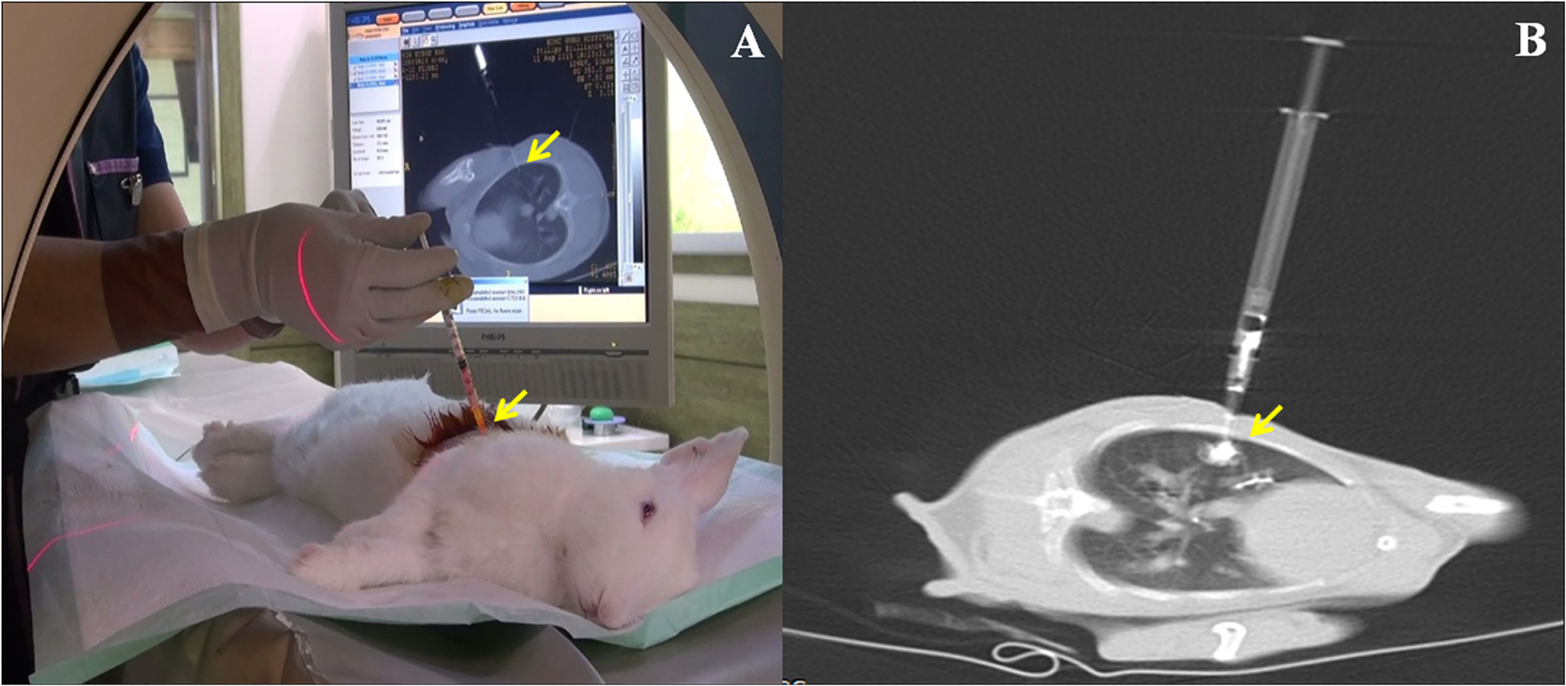
Real-time computed tomography (CT) fluoroscopy-guided implantation. (A) Real-time CT fluoroscopy-guided percutaneous inoculation in a rabbit lung. (B) VX2 carcinoma cell suspensions containing lipiodol were injected into the lung parenchyma under real-time CT fluoroscopy guidance. Arrows indicate the administered suspensions.

**Fig 2:**
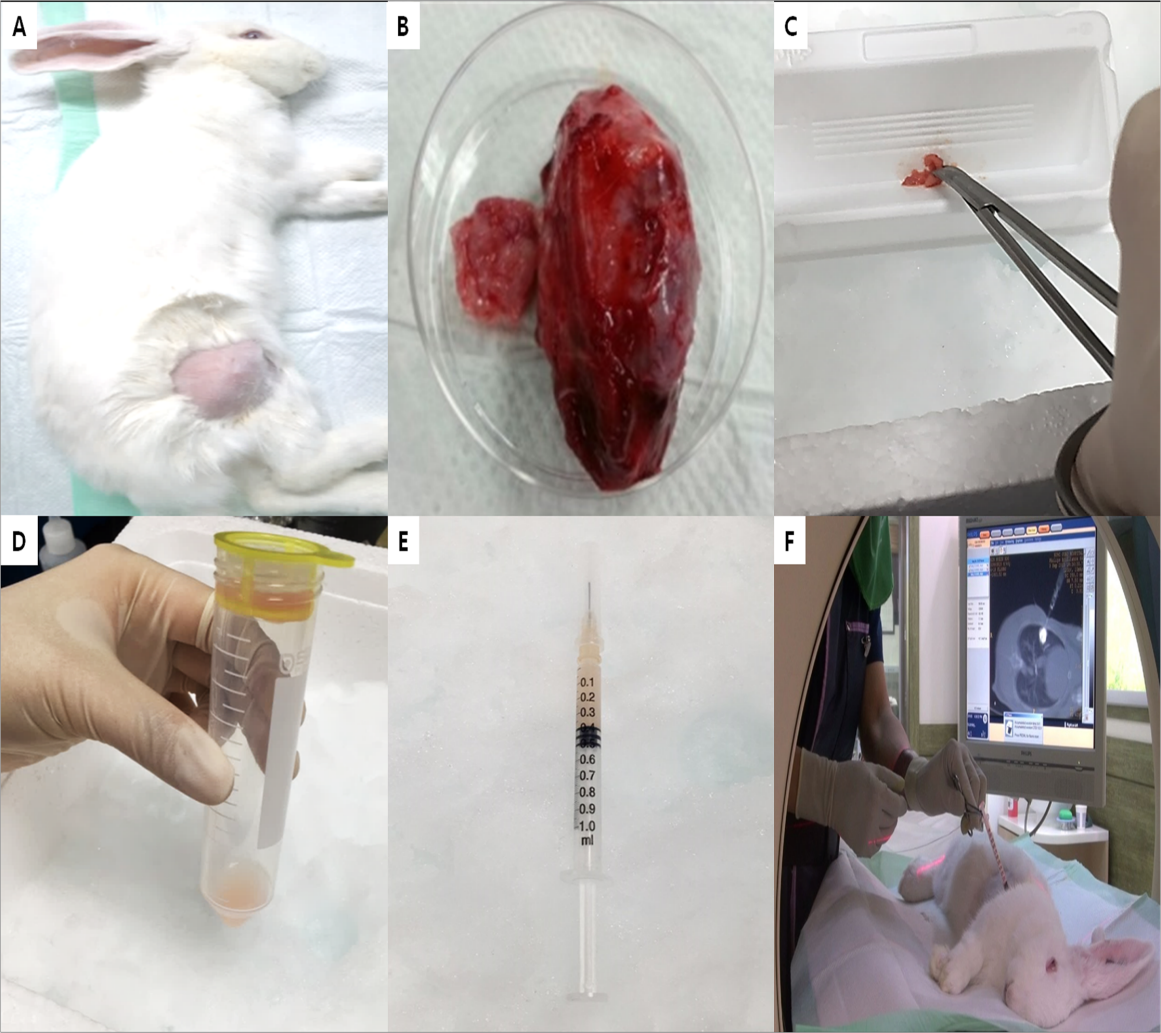
Summary of the rabbit model of VX2 lung cancer. (A) VX2 carcinoma in the hind leg of a rabbit. (B) Harvested VX2 carcinoma tissue. (C) Finely minced VX2 tissue. (D) VX2 carcinoma suspension after filtration through a 100-μm cell strainer. (E) Mixture of VX2 cells, lipiodol, and Matrigel for inoculation. (F) VX2 inoculation under real-time computed tomography fluoroscopy guidance.

After the administration of local anesthesia, the introducer needle was inserted into the peripheral lung region under CT fluoroscopy guidance (Figs 1 and 2). The suspension (450 μL–500 μL) was injected into the lung parenchyma, and the development of pneumothorax or hemothorax was immediately detected. The rabbits were then placed in individual cages in a temperature- and humidity-controlled room, with food and water available ad libitum. Twelve hours later, the rabbits received 0.3 mL (10 mg/kg) of the antibiotic enrofloxacin (Daewoong Pharmaceutical Co., Seoul, Korea) via intramuscular injection.

### ^18^F-fluorodeoxyglucose positron emission tomography imaging

Two weeks after VX2 inoculation, the lung tumors had reached a size of almost 30–40 mm, and were thus suitable for preclinical research [22]. To assess tumor formation in each group, 12 randomly selected rabbits (3 per group) underwent 18F-fluorodeoxyglucose positron emission tomography (^18^FDG-PET) using a Gemini TF 16-slice PET scanner (Philips Medical Systems, Cleveland, OH, USA). All rabbits were fasted for at least 12 hours before scanning and were anesthetized with tiletamine-zolazepam and xylazine as described above. Each rabbit received approximately 37 MBq (1 mCi) ^18^FDG through the auricular vein, and chest PET images were acquired over 60 min. PET images were displayed and analyzed at a dedicated workstation (Extended Brilliance Workspace 3.5; Philips Medical Systems).

### Necropsy and hematoxylin and eosin staining

On the day after the ^18^FDG-PET scanning, the rabbits were anesthetized with tiletamine-zolazepam and xylazine as described above and sacrificed via injection of 10 mL of air into the auricular vein. The anatomical and histopathological features (size, pleural leakage, and metastasis) of the tumors in the pleural cavity or lung were determined. The harvested tumor tissue was fixed in 10% formalin and embedded in paraffin. Tissue sections were then cut and examined pathologically by hematoxylin and eosin staining (Dako, Glostrup, Denmark).

## Results

### Real-time CT fluoroscopy-guided inoculation

**Fig 3:**
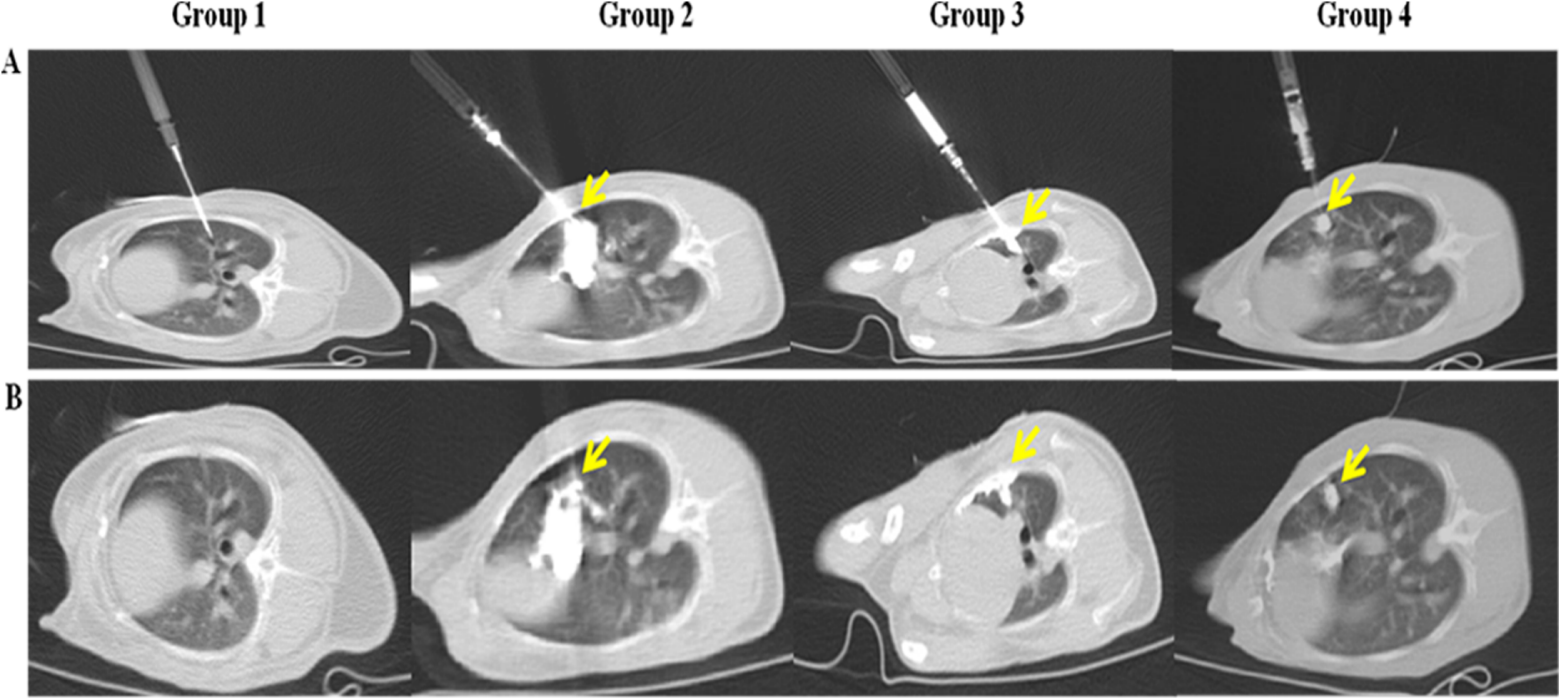
Real-time computed tomography (CT) fluoroscopy-guided percutaneous inoculation of VX2 tumor suspensions. (A) CT images during inoculation under CT fluoroscopy guidance. (B) CT images after inoculation. The tissue suspensions were not detectable in the images for group 1. In group 2, the tissue suspensions had substantially diffused throughout the lung parenchyma, pleural cavity, and thoracic wall. The cell suspensions in group 3 leaked into the pleural cavity via the inserted needle track. In group 4, the cell suspensions successfully formed globular structures, and there was no leakage. The yellow arrows indicate the suspensions.

During CT fluoroscopy-guided inoculation of the rabbits in group 1, the inserted needle was easily identified on the images, but whether the tissue suspension was injected into the lung parenchyma could not be clearly determined (Table 2, Fig 3). In contrast, the lipiodol-containing tissue suspensions were easily visualized and monitored via real-time CT fluoroscopy in all rabbits in group 2. However, during inoculation, a large amount of the tissue suspension leaked into the pleural space (five of five group 2 rabbits, 100%) or the lung parenchyma, pleural cavity, and thoracic wall (three of five group 2 rabbits, 60%). In fact, in two rabbits in group 2 (40%), the suspension entered the pleural cavity and thoracic wall without even entering the lung parenchyma.

**Table 2:** Comparison of the results of computed tomography (CT) fluoroscopy-guided inoculation and tumor necropsy.

Real-time CT fluoroscopy						Necropsy				
Group (n)	Suspension location (Incidence, %)					Tumor location (Incidence, %)				
	L	LP	PT	LPT	None	L	LP	PT	LPT	TW
1 (5)	0	0	0	0	5 (100)	0	2 (40)	1 (20)	1 (20)	1 (20)
2 (5)	0	0	2 (40)	3 (60)	0	0	0	0	5 (100)	0
3 (6)	4 (66.7)	2 (33.3)	0	0	0	4 (66.7)	2 (33.3)	0	0	0
4 (22)	22 (100)	0	0	0	0	22 (100)	0	0	0	0

L, lung; LP, lung and pleural cavity; PT, pleural cavity and thoracic wall; LPT, lung, pleural cavity, and thoracic wall; TW, thoracic wall at the injection site.

To prevent pleural leakage, the suspension volume was reduced from 500 μL to 450 μL in the group 3 rabbits, and Matrigel was added to gelate the mixture. The rabbits in this group were inoculated with a 20-gauge needle, as were the rabbits in group 2. Visualization via CT fluoroscopy revealed that the suspensions were localized in the lung parenchyma in globular masses (four of six group 3 rabbits, 66.7%) or had spread into the lung and pleural space via the track left by the needle (two of six group 3 rabbits). However, seeding in the thoracic wall was not observed in any of the rabbits in group 3.

It is possible that 18- and 20-gauge needles create a large pore at the injection site, through which the suspension leaks into the pleural cavity. Accordingly, we decided that these needle sizes were not appropriate for percutaneous inoculation in animal models of lung cancer animal. Therefore, we used a 26-gauge needle (outside diameter, 0.45 mm) instead of a 20-gauge needle (outside diameter, 0.914 mm) to inoculate the rabbits in group 4 (n = 22). Pleural cavity leakage via the inserted needle track was not observed in any of the rabbits in this group.

A small pneumothorax was observed in two of the total 38 rabbits (5.3%) on CT images during real-time CT fluoroscopy-guided inoculation. Both instances occurred in rabbits in group 1, and these rabbits survived until the end of the study without requiring any intervention.

### Patterns of tumor formation on PET/CT and necropsy

**Fig 4:**
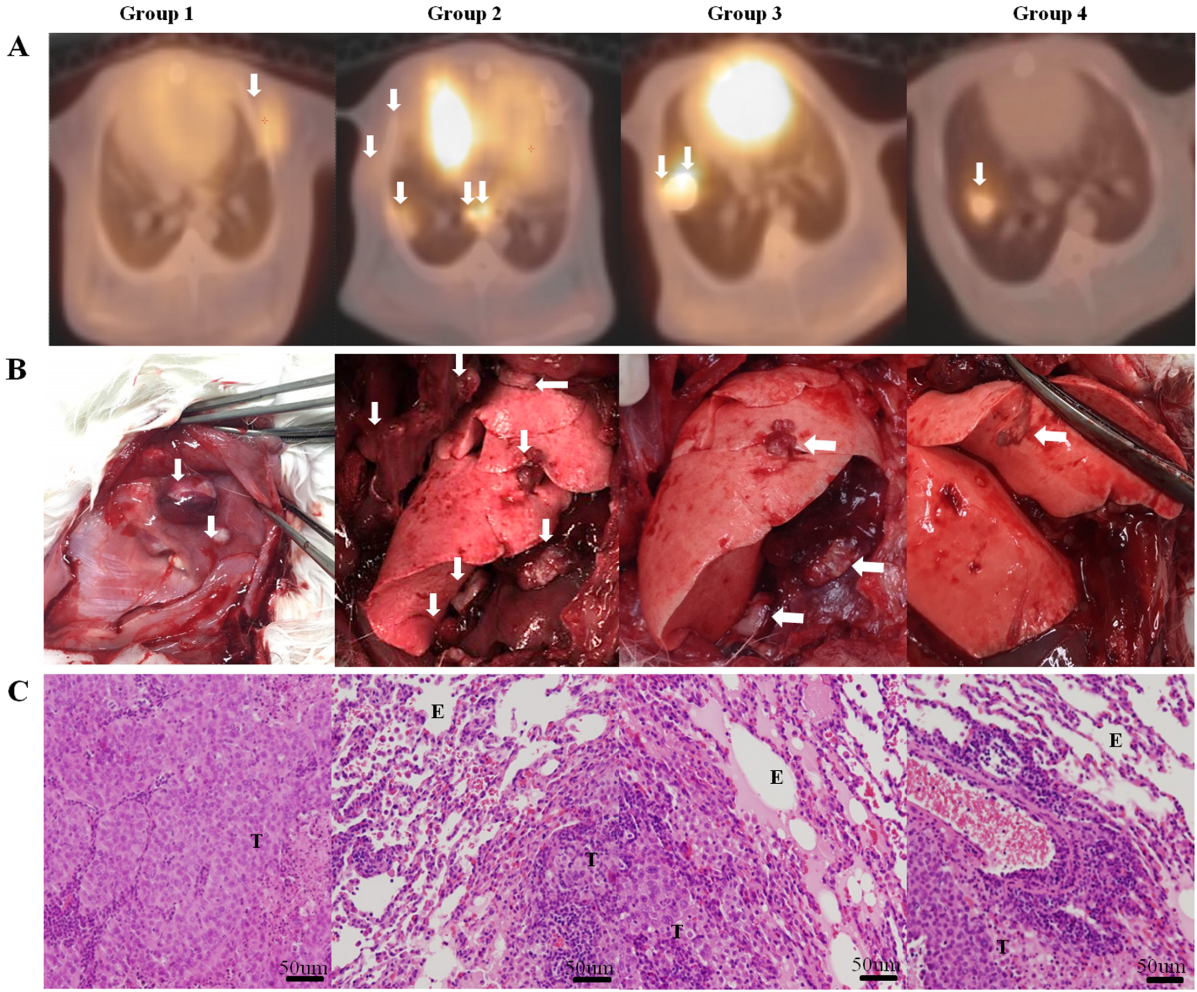
Results of lung cancer model in rabbit on positron emission tomography/computed tomography (PET/CT) scan, necropsy, and H&E staining. (A) Tumor images on PET/CT scan. (B) Tumor formation on necropsy. (C) Microscopic findings on H&E staining (×50). In group 1, tumors are detected in the thoracic wall on PET/CT and necropsy. In group 2, multiple tumor lesions are shown in the lung parenchyma, pleural cavity, and thoracic wall on PET/CT with necropsy. In group 3, hypermetabolic lesions are shown only in lung parenchyma and mild hyper-metabolism is adjacently detected to pleural cavity on PET/CT. Multiple tumor lesions are identified in the lung and plural cavity on necropsy. Solitary lung cancer models in rabbit were developed in group 4. The white arrow indicates the VX2 carcinoma lesion. Microscopic findings of tumor cell (T) and alveolar epithelial cell (E).

PET/CT scans of the group 1 rabbits showed ^18^FDG accumulation in the lung parenchyma and/or pleural cavity. Uptake of ^18^FDG was even observed in the thoracic wall, although the VX2 tissue suspensions were not visible on CT images during inoculation. Necropsy revealed that two of the five group 1 rabbits (40%) had multiple tumors in the lung parenchyma and pleural cavity. The others had VX2 tumor lesions in the lung, pleural cavity, and thoracic wall (one rabbit, 20%), in the pleural cavity and thoracic wall without lung involvement (one rabbit, 20%), and in the thoracic wall only, at the injection site (one rabbit, 20%) (Fig 4).

In group 2 rabbits, ^18^FDG uptake was detected in the lung parenchyma, pleural cavity, and thoracic wall at the injection site. On necropsy, VX2 tumors were present at all of these sites in all five group 2 rabbits. In rabbits in group 3, the PET/CT results mimicked the fluoroscopic CT-guided results: ^18^FDG accumulated mainly in the lung parenchyma and the adjacent pleural cavity. Necropsy revealed that four of the six group 3 rabbits (66.7%) developed localized VX2 tumors in the lung parenchyma, whereas two (33.3%) developed multiple VX2 tumors in both the lung and pleural cavity. However, there were no thoracic wall lesions in rabbits in this group. PET/CT scans of the group 4 rabbits showed solitary tumor formation in the lungs, similar to that shown by CT imaging. In all 22 rabbits in group 4, PET/CT and necropsy identified globule-shaped solitary VX2 carcinomas in the lung parenchyma only.

Microscopic examination revealed that the VX2 cancer cells had undergone coagulative necrosis. They formed irregularly shaped nests of viable cells with blurred nuclear chromatin. Microscopically, there were no noticeable differences in the cancer cells between the four groups.

## Discussion

We developed a novel technique for creating a solitary VX2 lung tumor in a rabbit lung cancer model. The technique involves inoculating a lipiodol- and Matrigel-containing tumor cell suspension into rabbits using a small needle under real-time CT fluoroscopy guidance (S1 Video). This new technique offers several advantages over previously reported techniques such as post-mini-thoracotomy percutaneous inoculation and bronchoscope-guided inoculation [22–24]. First, it enables a more precise localization of the VX2 tumor cell suspension: lipiodol is detectable on CT scans and Matrigel prevents the suspension from spreading. Second, our nonsurgical method has a lower risk of associated complications such as pleural effusion, inflammation, hemothorax, and pneumothorax than do surgical methods. All 38 rabbits in our study survived, although a small pneumothorax was observed in two rabbits. Third, it is possible to easily perform the tumor cell inoculation within a period of several minutes. Fourth, it is not necessary to provide artificial ventilation during inoculation.

Studies performing CT-guided (rather than real-time CT fluoroscopy-guided) VX2 tumor cell inoculations with or without Matrigel and with 18- or 21-gauge needles have reported successful formation rates of VX2 lung cancer of 45.8–77.8%; however, multiple rather than solitary lesions were obtained [25–27]. Similar to the procedures performed in previous studies, we first percutaneously injected VX2 tissue suspensions without Matrigel using an 18-gauge needle under real-time CT fluoroscopy guidance (group 1). On necropsy, multiple cancer lesions were observed in the lung parenchyma, pleural cavity, and/or thoracic wall. To improve targeting, we then mixed the cell suspension with the radiopaque dye lipiodol and used a 20-gauge needle (group 2). These modifications allowed clear visualization of the cell suspension via CT fluoroscopy, which showed it spreading from the lung parenchyma into the pleural space and thoracic wall. Necropsy findings for rabbits inoculated in this manner were similar to those of previous studies [23–27].

In the next trial, we used Matrigel, a gelatinous protein, to gelate the suspension to prevent leakage; lipiodol was also added to the suspension (group 3). In addition, we reduced the tumor cell volume from 500 μL to 150 μL to decrease the total suspension volume to 450 μL. CT fluoroscopic images showed globular suspensions localized in the lung parenchyma, although in some rabbits, the suspensions had spread into the pleural space through the 20-gauge needle track. In an attempt to prevent spreading, we reduced the needle size to 26-gauge.

To avoid clogging the needle with tissue debris, we finely minced the VX2 tumor tissues and filtered them through a 100-μm cell strainer to produce a single-cell VX2 suspension, which contained both Matrigel and lipiodol (group 4). Doing so resulted in successful leakage-free injection. Globular focal ^18^FDG accumulation in the lung parenchyma was observed on PET/CT images, and solitary VX2 lung cancer nodules developed in all group 4 rabbits 2 weeks later, as determined via necroscopy.

In conclusion, we have developed a new rabbit lung cancer model of solitary VX2 lung tumors. Inoculation of a tumor cell suspension including lipiodol and Matrigel through a small needle under real-time CT fluoroscopy is an easy and safe method for the successful formation of VX2 solitary lung tumors in rabbits. Moreover, this model will be useful in surgical practice, fluorescence cancer imaging with indocyanine green, and interventional radiology studies. We recommend the following protocol for creating solitary VX2 lung tumors in rabbits: 1) tumor tissue should be finely chopped and filtered through a 100-μm cell strainer to enable tumor cell suspension inoculation through a small needle; 2) the tumor cells should be mixed with a radiopaque dye and Matrigel (ratio, 1:1:1; total volume, <450 μL) to avoid leakage; and 3) a small needle such as a 26-gauge needle should be used.

One limitation of our study is that VX2 carcinoma is not relevant to human cancer. However, the rabbit VX2 solitary lung tumor model is useful and effective for evaluating and developing surgical and interventional techniques in preclinical studies. Another limitation is that real-time CT fluoroscopy is not available in most laboratories. However, most hospital laboratories, where research using this model is usually conducted, have CT and/or PET equipment, and it is not difficult to perform CT-guided VX2 lung cancer modeling in translational research laboratories.

## Supporting information

S1 Video Real time CT fluoroscopy guided VX2 injection. (MP4)
