# Correction: Real-time computed tomography fluoroscopy-guided solitary lung tumor model in a rabbit

**DOI:** 10.1371/journal.pone.0192527

**Published:** 2018-02-02

**Authors:** The PLOS ONE Staff

The fourth and eighth authors’ names are spelled incorrectly. The publisher apologizes for the errors. The correct names are: Ji Yun Rho and Hyun Koo Kim. The correct citation is: Choi BH, Young HS, Quan YH, Rho JY, Eo JS, Han KN, et al. (2017) Real-time computed tomography fluoroscopy-guided solitary lung tumor model in a rabbit. PLoS ONE 12(6): e0179220. https://doi.org/10.1371/journal.pone.0179220.
